# The effects of different extraction methods on essential oils from orange and tangor: From the peel to the essential oil

**DOI:** 10.1002/fsn3.3785

**Published:** 2023-10-25

**Authors:** Min Kyung Park, Ji Yoon Cha, Min‐Cheol Kang, Hae Won Jang, Yun‐Sang Choi

**Affiliations:** ^1^ Food Processing Research Group Korea Food Research Institute Wanju Korea; ^2^ Department of Food Science and Biotechnology Sungshin Women's University Seoul Korea

**Keywords:** citrus fruits, citrus peel, cold‐pressing, distilled steam extraction, essential oil, flavors

## Abstract

Citrus fruits are largely consumed due to their unique and pleasant aromas. *Citrus* hybrids have been developed to enhance their flavors and bioactivities. Citrus aroma depends on the composition of the volatile compounds in citrus essential oils (CEOs), which are mostly located in the peels. During the extraction of CEOs, a specific series of chemical reactions occurred depending on the extraction methods (CP, cold pressing; HD, hydrodistillation), leading to variations in the composition of volatile compounds. In this study, the orange and the tangor which is a hybrid between *C. reticulata* × *C. sinensis* were investigated to compare the changes in volatile compounds based on the extraction methods. Results showed that the CP‐specific volatile compounds were sesquiterpenes, oxygenated monoterpenes, and fatty acid derivatives, while the HD‐specific volatile compounds were terpinyl cation derivatives, limonene, and 4‐vinylguaiacol. On the other hand, the contents of some volatile compounds ((E)‐ocimene, α‐terpinene, and α‐terpinolene) were affected by the *Citrus* species rather than by the extraction methods. In particular, during HD, terpinene‐4‐ol and 4‐vinylguiacol, known as off‐flavor compounds in citrus juice, were formed more abundantly in the orange than in the tangor. In conclusion, these results provide comprehensive data on essential oils, especially those derived from oranges and tangors, for selecting the appropriate extraction method for obtaining a higher yield and quality of citrus flavor.

## Graphical abstract

This study investigated the changes of the volatile compounds in orange and tangor peels and the essential oils prepared using different extraction methods: hydrodistillation and cold pressing. We provide comprehensive data on essential oils, especially those derived from oranges and tangors, to aid in selection of the appropriate extraction method for obtaining a higher yield and quality of citrus flavor.

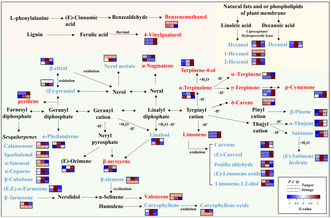

## 1 INTRODUCTION

Citrus flavors are widely used in various industrial fields, such as beverages, cosmetics, and perfumes, due to their unique and pleasant aromas. *Citrus*, which belongs to the Rutaceae family, includes diverse *Citrus* species, such as *C. sinensis* (orange), *C. reticula* (mandarin), *C. limon* (lemon), *C. paradise* (grapefruit), and *C. junos* (yuzu). The characteristic flavor differences between citrus species have been studied by analyzing volatile compounds using a gas chromatography–mass spectrometry (GC–MS; Elmaci & Altug, 2005; Hou et al., 2020) or by calculating odor activity values (OAVs) of the flavor components using the combined technology of olfactory sensation and instrument analysis (Deterre et al., 2016; Feng et al., 2018).

Terpenes, which consist of *n*‐isoprene units, are the main volatile compounds in plant essential oils, including numerous types of *Citrus* species. Monoterpenes (two isoprene units) are sometimes described to mainly have a note of leaf‐like odor, while sesquiterpenes (three isoprene units) generally have a note of flower‐like odor (Dudareva et al., 2006). Limonene, which has a pleasant and citrus‐like odor (Rodríguez et al., 2017), is the main monoterpene found in numerous citrus oils. It comprises up to 50% of the volatile compounds in citrus fruits (Dugo & Di Giacomo, 2002). Citrus oils are mostly composed of terpenes with a small percentage of oxygenated terpenoids (such as aldehydes, esters, and alcohols) that have a strong and pleasant olfactory impact (Dupuy et al., 2011). In addition, volatile compounds derived from fatty acids (such as aldehydes, esters, and alcohols) are strongly related to citrus‐like odor attributes. For example, linalool and citronellol contribute to fruit/flower odor description, while octanal, decanal, ethyl hexanoate, and ethyl octanoate are related to green‐like odor description (Jia et al., 2022). These volatile compounds can be easily degraded or oxygenated by some factors, such as headspace oxygen, enzymes, and thermal treatment (Rouseff & Naim, 2000). In particular, thermal treatment easily induces a series of chemical reactions and leads to the decomposition of citrus‐active volatile compounds or the formation of off‐flavor compounds. For example, α‐terpineol and d‐carvone, which are considered to be off‐flavor compounds, are generated through the acid‐catalyzed degradation of limonene (Vervoort et al., 2012).

Citrus essential oil (CEO) is a concentrated organic soluble plant extract that has been used for various purposes, such as producing perfumes, cosmetics, and medicines. The aroma quality, biological effects (Bouabdallah et al., 2022), and yield of CEOs can be determined by the extraction methods. CEOs are mainly obtained using distillation (hydrodistillation, steam distillation, and dry distillation) and nonthermal extraction (cold pressing, supercritical CO_2_ fluid extraction, and solvent extraction; Filly et al., 2016). In addition, combined extractions, such as a solvent‐free microwave extraction (SFME) and an ultrasound‐assisted extraction, have been applied to obtain high yields of CEOs and prevent changes in citrus flavors (Chemat et al., 2020; Nayak et al., 2015). The volatile compound profiles of CEOs are highly dependent on the extract method. Among several extraction methods for citrus fruits, cold pressing (CP) and hydrodistillation (HD) are considered conventional. During HD, citrus peel is exposed to boiling water and releases essential oils via water evaporation. Although there is a high yield of CEOs, there is a loss or degradation of volatile compounds due to the long extraction times and elevated temperatures (Bayramoglu et al., 2008). When CP is applied, the peels are vigorously agitated with water. Although CP has a relatively lower yield of volatile compounds than HD, it is preferred because it preserves the citrus‐like aroma as much as possible. However, during agitation, air is thrashed into the liquid, creating conditions favorable for hydrolysis, oxidation, and resinification (Ferhat et al., 2016).

The aim of this study was to investigate changes in the volatile compound profiles of the orange and tangor essential oils extracted using different extraction methods (CP and HD). Orange is the most widely consumed citrus fruit worldwide, and its flavors have been investigated over the last few decades. Hybrids between mandarin and sweet orange and their descendants generally show similar fruit sizes, shapes, colors, and flavor features somewhere between those of the parents (Yu et al., 2015). Tangor (*C. reticulata* × *C. sinensis*) is a *Citrus* hybrid of mandarin and orange. The tangor used in this study, which is called as “golden aroma,” is mostly consumed in South Korea and Japan and has a stronger sweetness and citrus‐like aroma than oranges. To investigate the characteristic volatile compounds of the tangor essential oil and the changes in volatile compound profiles depending on the extraction method, orange, which is consumed globally, was used as a control for comparison. In addition, because CEOs are mainly located in citrus fruit peels, each citrus peel was investigated to obtain information on its characteristic citrus aroma.

## 2 MATERIALS AND METHODS

### 2.1 Chemicals and reagents

All chemicals, including methanol and 3,4‐dimethylphenol, were purchased from Sigma‐Aldrich.

### 2.2 Plant material

Citrus fruits, including orange (*Citrus sinensis*) and tangor (*Hwanggeumhyang*, *C. Beni Madonna*) were applied to investigate the changes of volatile compound profiles according to different extraction methods. The orange, which was imported from the United States, was purchased from a local market in South Korea, and the tangor originated from Jeju Island, South Korea. After washing with water, the peels were obtained with the albedo (white inner membrane) removed and maintained at 4°C before analysis.

### 2.3 Hydrodistillation (HD)

The peels of citrus fruits (500 g) were placed in a distillation flask along with distilled water (1.5 L). The flask was heated for 3 h to produce steam which carries the volatile essential oil from the peel of citrus fruits. The steam and essential oil vapors traveled through a cooled condenser. The condensed mixture of water and essential oil was collected in a receiving flask. The supernatant, essential oil, was separated from the water by centrifugation (13,008 *g*, at 4°C for 10 min).

### 2.4 Cold pressing (CP)

In brief, the peels were pressed at room temperature by juicer (Angelia 7000P; Angel Juicer). The mixture of emulsion and oil was separated by centrifugation (13,008 *g*, at 4°C for 10 min). The supernatant was collected and maintained at −18°C before analysis.

### 2.5 Volatile compound analysis

The volatile compounds in the peel and essential oils were analyzed using gas chromatography–mass spectrometry detector (GC–MSD) system coupled with solid‐phase microextraction (SPME) method. The peels (1.0 g) or 100 μL of the essential oil and the internal standard (2 μL, 3,4‐dimethylphenol, 100 mg/L in methanol) were placed into a 20‐mL amber headspace vial. Divinylbenzene/carboxen/polydimethylsiloxane/fiber (DVB/CAR/PDMS, 50/30 μm; Supelco) was inserted into the headspace of vial for 30 min at 40°C, then the volatile compounds were thermally desorbed in the injector port of GC for 5 min at 230°C.

7890A Agilent GC system (Agilent Technologies) and 5975C MSD mass detector (Agilent Technologies) were applied coupled with a DB‐WAXUI capillary column (30 m × 0.25 mm i.d. × 0.25 μm film thickness; J&W Scientific). The conditions of GC–MS were as follows: carrier gas Helium (99999%); gas flow rate 0.8 mL/min; split ratio 50:1; injector and transfer 230°C and 250°C; oven temperature initial temperature started at 40°C for 5 min, increased to 200°C with a rate of 4°C per min, and maintained for 2 min. The volatile compounds were obtained by the electron impact (EI) ion source at 70 eV and scanned in the range of 35–350 atomic mass units (a.m.u.). The volatile compounds were identified by comparing retention times and mass spectral data with those of authentic standard compounds or commercial GC–MS library (Wiley7.0, W9N08). The retention indices (RI) of volatile compounds were determined using n‐alkane (C_7_ to C_22_). The amount of each volatile compound was determined by comparing its peak area to that of an internal standard. Then, the relative amounts of each volatile compound were expressed as a percentage of the total content. All experiments were conducted in triplicate.

### 2.6 Statistical analysis

Analysis of variance (ANOVA) was conducted using IMB SPSS Statistics for Windows, version 25.0 (IBM Corp.) to evaluate statistical changes depending on different extract methods. The result of Duncan's multiple range test was presented at a significant different level (*p* < .05). Orthogonal partial least squares‐discriminant analysis (OPLS‐DA) was conducted to discriminate two different coffees on the basis of volatile compound profiles using SIMCA 16 (Umetrics). Heat map visualization based on the *z*‐score distribution was shown using a heatmap.2 function in the gplot package implemented in R environment (version 4.0.4).

## 3 RESULTS AND DISCUSSIONS

In this study, over 98% of the total amount of volatile compounds were detected using GC–MS and identified by comparing their mass spectra with those of authentic chemical compounds or commercial mass spectrum libraries. Table S1 lists identified the volatile compounds, relative peak areas, and RIs. In total, 107 volatile compounds were identified across all peel and essential oil samples, including 17 monoterpene hydrocarbons (monoterpenes), 29 oxygenated monoterpenes, 10 sesquiterpene hydrocarbons (sesquiterpenes), 5 oxygenated sesquiterpenes, 14 aliphatic hydrocarbons and derivatives, 7 cyclic or aromatic hydrocarbons, and 1 heteroaromatic hydrocarbon in the tangor and including 18 monoterpene hydrocarbons (monoterpenes), 30 oxygenated monoterpenes, 8 sesquiterpene hydrocarbons (sesquiterpenes), 5 oxygenated sesquiterpenes, 23 aliphatic hydrocarbons and derivatives, 7 cyclic or aromatic hydrocarbons, and 2 heteroaromatic hydrocarbons in the orange. When compared according to the extraction method, the detection of cyclic or aromatic hydrocarbons increased during HD extraction, while the detection of oxygenated sesquiterpenes and aliphatic hydrocarbons increased during CP extraction, regardless of *Citrus* species. Based on the results, the detection of oxygenated volatile compounds significantly increased in CP. Since they have distinctive odor characteristics, it might be assumed that the changes in the composition of volatile compounds may have influenced the quality of the samples. Further details were discussed in the following section. The results showed that monoterpene hydrocarbons, including limonene, accounted for 97.3%–99.6% of the total amount of volatile compounds detected as reported in previous studies (Dugo & Di Giacomo, 2002; Erasto & Viljoen, 2008). Citrus peels and essential oils contained a high content of limonene (85.5%–93.1%). Limonene is a major constituent of citrus peels and is widely used as a flavor and fragrance additives due to its citrus‐like odor. In addition, it can be used as a precursor for various monoterpenoids, which affect flavor characteristics and biological properties (antimicrobial, antioxidant, and anti‐inflammatory activities; Erasto & Viljoen, 2008). In this study, the content of volatile compounds, including limonene, varied depending on the citrus species and extraction method used.

### 3.1 Comparison of volatile profiles between orange and tangor

**FIGURE 1:**
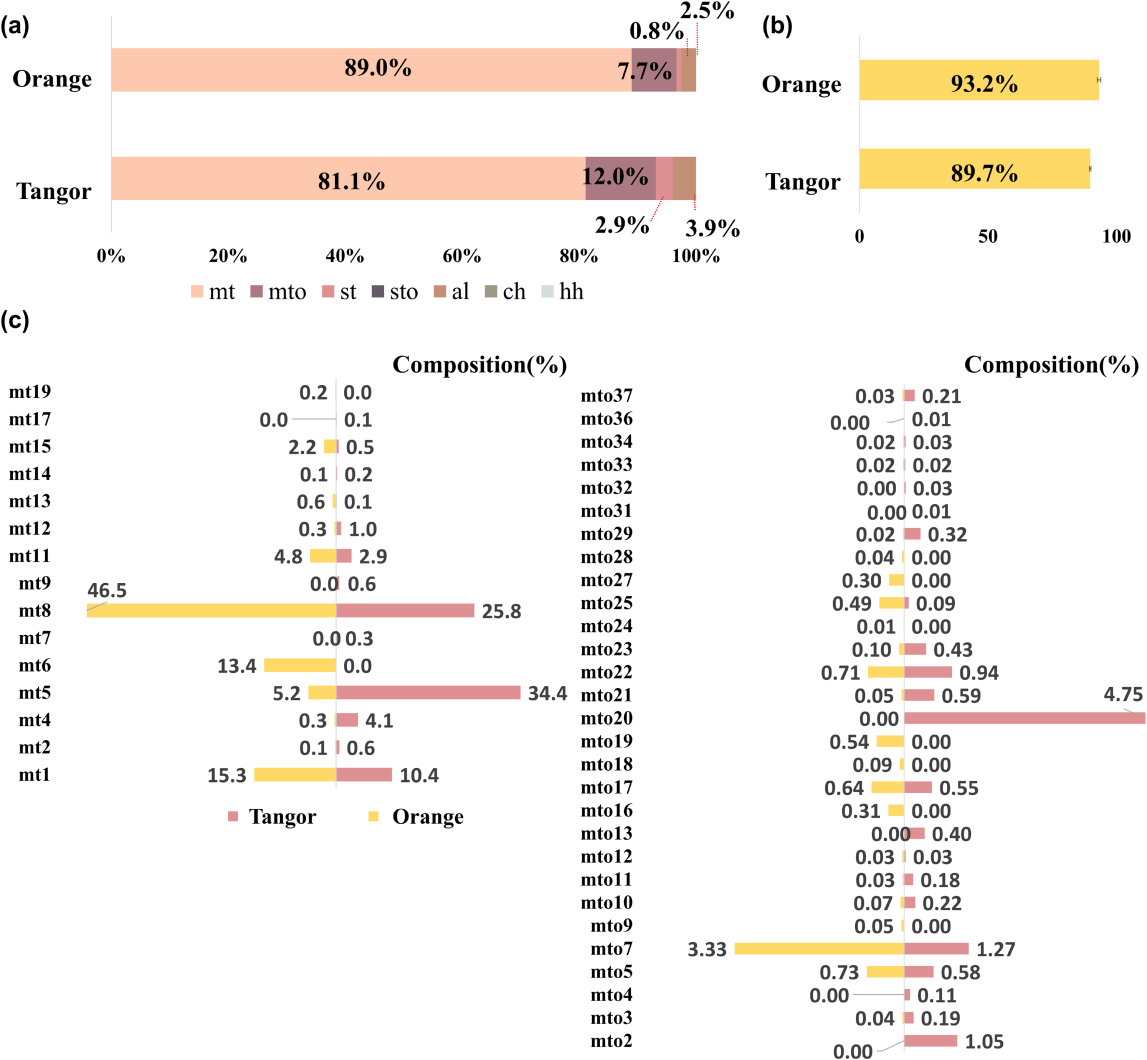
The composition ratio of volatile compounds, which were classified by chemical group, (a) and the content of limonene (b) in orange and tangor. The comparison of composition ratio of volatile compounds, belonging to monoterpene hydrocarbons and oxygenated monoterpenes (c). Abbreviations were as follows: al, aliphatic hydrocarbons; ch, cyclic or aromatic hydrocarbons; hh, heteroaromatic hydrocarbons; mt, monoterpene hydrocarbons; mto, oxygenated monoterpenes; st, sesquiterpene hydrocarbons; sto, oxygenated sesquiterpenes.

Citrus essential oils are accumulated in secretory cavities scattered throughout the flavedo layer of citrus fruits (Ahmad et al., 2006). Therefore, flavedo peels obtained from the orange and the tangor were investigated to compare the flavor characteristics of each citrus fruit. Figure 1 summarizes the comparison of the volatile compound profiles between the orange and the tangor. The results showed that limonene accounted for 93.2% and 89.7% of the total volatile compounds in the orange and tangor, respectively (Figure 1a). The proportion of limonene was relatively higher in the orange than tangor, and no significant differences were observed. As shown in Figure 1b, the composition of volatile compounds classified by chemical groups, excluding limonene, was expressed using a cumulative bar chart. The composition ratio of volatile compounds, excluding limonene, was compared because of its excessively high proportion among the total volatile compounds. Monoterpenes, oxygenated monoterpenes, and aliphatic hydrocarbons (aldehydes, alcohols, acids, and esters) accounted for the majority of volatile compounds in both citrus fruits. Monoterpenes showed a higher proportion of total volatile compounds in the orange than in the tangor, while oxygenated monoterpenes and aliphatic hydrocarbons accounted for higher proportions in the tangor. Since monoterpenes and oxygenated monoterpenes accounted for 93%–96% of the total volatile compounds, main volatile compounds within monoterpenes and oxygenated monoterpenes between the orange and the tangor were compared. Figure 1c presents a detailed bar graph comparing the composition ratios of each monoterpene hydrocarbon and oxygenated monoterpene. β‐Myrcene (mt8), δ‐carene (mt6), α‐terpinolene (mt9), β‐phellandrene (mt11), α‐pinene (mt1), linalool (mto7), α‐citral (mot19), and (Z)‐geraniol (mto25) accounted for a higher proportion of the totals in the orange compared to the tangor. In tangor, sabinene (mt5), β‐pinene (mt4), (E)‐limonene oxide (mto2), carvone (mto20), and geranyl acetate (mto21) showed a significant difference compared to the orange. The results of this study were similar to those of previous studies. Deterre et al. (2016) reported that α‐pinene, β‐myrcene, δ‐carene, α‐terpinolene, and linalool were main volatile compounds contributing to the orange‐like aroma. In addition, sabinene, myrcene, octanal, α‐terpineol, and α‐pinene were the main volatile compounds in novel tangors, followed by limonene and linalool (Azevedo et al., 2019).

The OPLS‐DA approach was performed to discriminate between the orange and the tangor based on the identified volatile compounds. The OPLS‐DA models showed appropriate modeling and predictive abilities using one predictive component and two orthogonal components (R^2^Y = 0.998, Q^2^ = 0.995). Based on the values of projection of variable importance (VIP) and correlation coefficient (*pcorr*), 64 variants were selected to determine the flavor characteristics of each sample (Table 1, VIP > 1 and I*pcorr*I > 0.6). A VIP value larger than 1 is considered influential for the separation between groups in the score plots generated from the OPLS‐DA analysis (Lenz & Wilson, 2007). Table 1 lists the important variables used to discriminate between the orange and the tangor. In the orange peel, the relative content of monoterpenes was higher than the tangor, while the contents of sesquiterpenes and oxygenated terpenes were higher in the tangor peel compared to the orange peel. Previous studies have reported that myrcene, linalool, valencene, and geraniol are important compounds in oranges (Espina et al., 2011; Hosni et al., 2010), while limonene, γ‐terpinene, limonene oxide, carvone, carvol, and β‐pinene are the main volatile compounds in mandarin (Espina et al., 2011; Hosni et al., 2010). In this study, these volatile compounds were also shown to be important for distinguishing between orange and tangor.

**TABLE 1:** Discriminant volatile compounds between orange and tangor.

No.	VIP	Pcorr	Volatile compound	No.	VIP	Pcorr	Volatile compound
*Orange specific*				*Tangor specific*			
mto27	1.02	1.00	(E)‐Geraniol	mt12	1.02	−1.00	γ‐Terpinene
al1	1.02	1.00	Hexanal	mto3	1.02	−1.00	(E)‐Sabinene hydrate
mto18	1.02	1.00	Nerol acetate	al16	1.02	−1.00	Acetic acid, nonyl ester
al15	1.02	1.00	1‐Octanol	al3	1.02	−1.00	Octanal
mto19	1.02	1.00	Citral	mt5	1.02	−1.00	Sabinene
mto9	1.02	1.00	Isopulegol	mto13	1.02	−1.00	(E)‐2‐Cyclohexen‐1‐ol, 1‐methyl‐4‐(1‐methyletheny)
mt6	1.02	1.00	delta‐Carene	mto2	1.02	−1.00	(E)‐Limonene oxide
st9	1.02	1.00	Valencene	st7	1.02	−1.00	β‐Farnesene
mt13	1.02	1.00	(E)‐Ocimene	mto20	1.02	−1.00	Carvone
mto28	1.02	1.00	Limonen‐10‐yl acetate	mto37	1.02	−1.00	Limonene‐1,2‐Diol
mt16	1.02	1.00	Neoalloocimene	mt2	1.02	−1.00	α‐Thujene
mt8	1.02	1.00	β‐Myrcene	mt9	1.02	−1.00	α‐Terpinene
mt19	1.02	1.00	p‐Cymene	mto21	1.02	−1.00	Geranyl acetate
mto25	1.02	1.00	(Z)‐Geraniol	mto11	1.02	−1.00	Dihydrocarvone
mt15	1.02	1.00	α‐Terpinolene	st8	1.02	−1.00	Eremophilene
al4	1.02	1.00	1‐Hexanol	mt17	1.02	−1.00	Perillene
mto7	1.02	1.00	Linalool	mto4	1.02	−1.00	Citronellal
al2	1.02	1.00	2‐Hexenal	mto31	1.02	−0.99	Perillyl Acetate
mto24	1.02	1.00	γ‐Geraniol	st12	1.02	−0.99	Calamenene
al11	1.02	0.99	Cosmene	al14	1.02	−0.99	Decanal
mto16	1.01	0.99	β‐Citral	st2	1.02	−0.99	β‐Cubebene
ch6	1.00	0.98	Benzenemethanol	al19	1.02	−0.99	2,6‐Octadiene, 2,6‐dimethyl‐
				st1	1.02	−0.99	α‐Copaene
				hh1	1.01	−0.99	α‐Naginatene
				mto29	1.02	−0.99	Carveol
				sto3	1.02	−0.99	Spathulenol
				st10	1.02	−0.99	(E,E)‐α‐Farnesene
				mto8	1.02	−0.99	Pinocarvone
				st5	1.02	−0.99	Caryophyllene
				mto36	1.02	−0.99	Cuminol
				ch3	1.02	−0.99	Benzaldehyde
				al6	1.01	−0.99	Nonanal
				sto5	1.01	−0.98	α‐sinensal
*Orange specific*				*Tangor specific*			
				st11	1.01	−0.98	delta‐Cadinene
				al23	1.01	−0.98	Octanoic acid
				mt7	1.01	−0.98	α‐Phellandrene
				mto23	1.01	−0.98	Perilla aldehyde
				st4	1.01	−0.98	β‐elemene
				mto10	1.01	−0.97	4‐Terpineol
				mt4	1.01	−0.97	β‐Pinene
				sto1	1.01	−0.97	Caryophyllene oxide
				mt14	1.01	−0.97	m‐Cymene

### 3.2 The changes in volatile profiles based on different extract methods

Citrus essential oils are primarily composed of highly volatile compounds that are reactive to oxygen, heat, or light (Mahato et al., 2019). In this study, cold‐processing (nonheated) and hydrodistillation extraction (heated) methods were compared to investigate the effects of thermal treatment on the formation of volatile profiles in the orange and the tangor.

**FIGURE 2:**
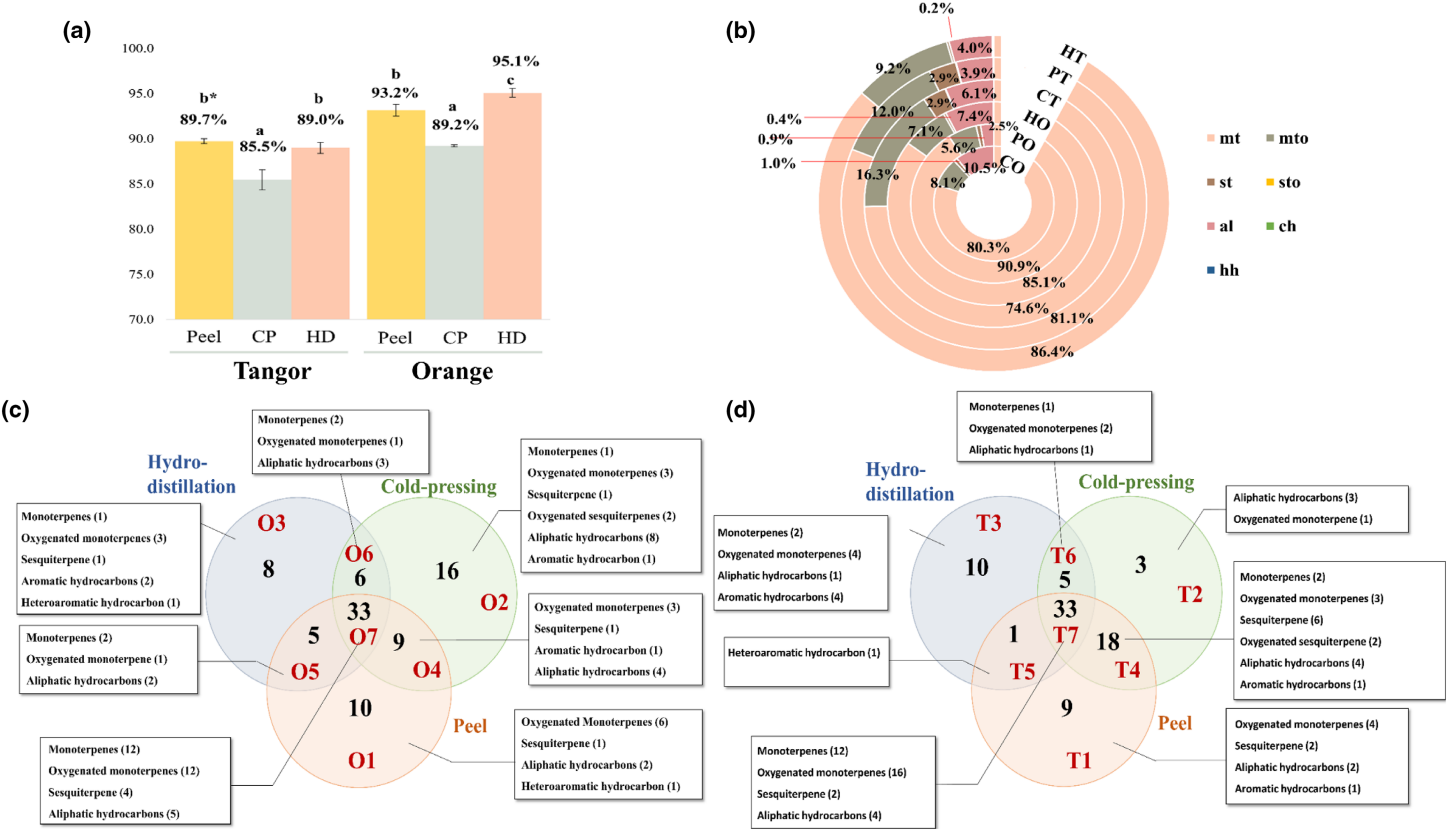
The summary of the changes of volatile compounds depending on the extraction methods for the orange and tangor essential oils. (a) Bar chart for the content of limonene. (b) Doughnut chart showing the distributions of the volatile compounds except for limonene. Identification of differentially expressed volatile compounds classified by chemical group depending on different extract method: Venn diagram of the differentially expressed volatile compounds in (c) orange and (d) mandarin (the number in each circle represent the amount of differentially expressed volatile between the different comparisons).

Figure 2 shows an overview of the changes in the volatile compound profiles derived from the orange and the tangor, which varied depending on the extraction method of the essential oil. Limonene is an important volatile compound due to its citrus‐like flavor and bioactivity (Rodríguez et al., 2017). As shown in Figure 2a, the composition of limonene, which is found in citrus peels, was lower in CP essential oils. This might be correlated with an increase in oxygenated monoterpenes levels. During CP, oxidation occurs easily because the sample is agitated with air. In particular, the content of limonene oxide, which is an oxygenated form of limonene, increased when CP was applied. Monoterpenes accounted for the highest content of the volatile compounds identified in this study. Since limonene accounted for >90% of the total volatile compounds, it was difficult to confirm the changes in other volatile compounds on the chart. Therefore, to compare the changes depending on the extraction method, the composition of the volatile compounds, excluding limonene, was represented in circle chart (Figure 2b). Monoterpenes were the major volatile components in both citrus peels and essential oils, followed by oxygenated monoterpenes, aliphatic aldehydes, and sesquiterpenes. In the tangor, compared to the peel, the content of oxygenated and aliphatic aldehydes increased in the essential oil extracted using CP, while that of oxygenated monoterpenes decreased in the essential oil extracted using HD. Similar changes in the chemical composition of tangor were observed in oranges. Oxygenated monoterpenes and aliphatic aldehydes were more abundant in essential oils extracted using HD than in the peel. However, less variability was observed when converting essential oil from the peel compared to that from the tangor.

To investigate the relationship between the volatile compound profiles and extraction methods, the number of volatile compounds that were overlapping between the peel and different essential oils was illustrated using a Venn diagram (Figure 2c,d). Regardless of the extraction method used, both volatile compounds derived from the citrus peel (O7 and T7) and those that were not transferred from the citrus peels (O1 and T1) into the essential oils were found. Monoterpene hydrocarbons were mostly transferred from the peel to the essential oil. Certain volatile compounds, such as oxygenated monoterpenes, sesquiterpenes, aliphatic hydrocarbons, and heteroaromatic hydrocarbons, were not detected in the essential oil and were only present in the peel. In this study, alloaromadendrene and geranyl acetate were detected only in the orange and tangor peels. Alloaromadendrene was reported as the one of main volatile compounds in the leaves of bitter orange (Ahmad et al., 2006). Geranyl acetate, which has a floral‐rosy odor (Jirovetz et al., 2006), has been found in citrus peels, leaves, and blooms (Darjazi, 2011). On the other hand, certain volatile compounds were not found in the citrus peels (O2, O3, O6, T2, T3, and T6), while some volatile compounds, which were contained in the citrus peel, were specific depending on the extraction method (O4, O5, T4, and T5). During the CP, the conditions can be created where hydrolysis or oxidation might occur (Ferhat et al., 2016). O2 and T2, which included volatile compounds that were only detected in the essential oils prepared using CP, included oxygenated volatile compounds, such as aldehydes (hexanal and dodecanal), alcohols (3‐hexen‐1‐ol, 2‐hexen‐1‐ol, and 1‐decanol), oxygenated compounds (limonene oxide), and esters (butanoic acid, hexyl ester, and acetic acid‐2‐ethylhyxyl ester) derived from fatty acids. In O3 and T3, cryptone, (E)‐carveol, 1,4‐Dimethyl‐4‐acetylcyclohexene, and 2‐methyoxy‐4‐vinylphenol were shown in both the essential oils prepared using HD. In particular, 2‐methoxy‐4‐vinylhenol (4‐vinylguaiacol) was reported to be the main contributor to the thermally induced off‐flavor of orange juice (Averbeck & Schieberle, 2011).

**FIGURE 3:**
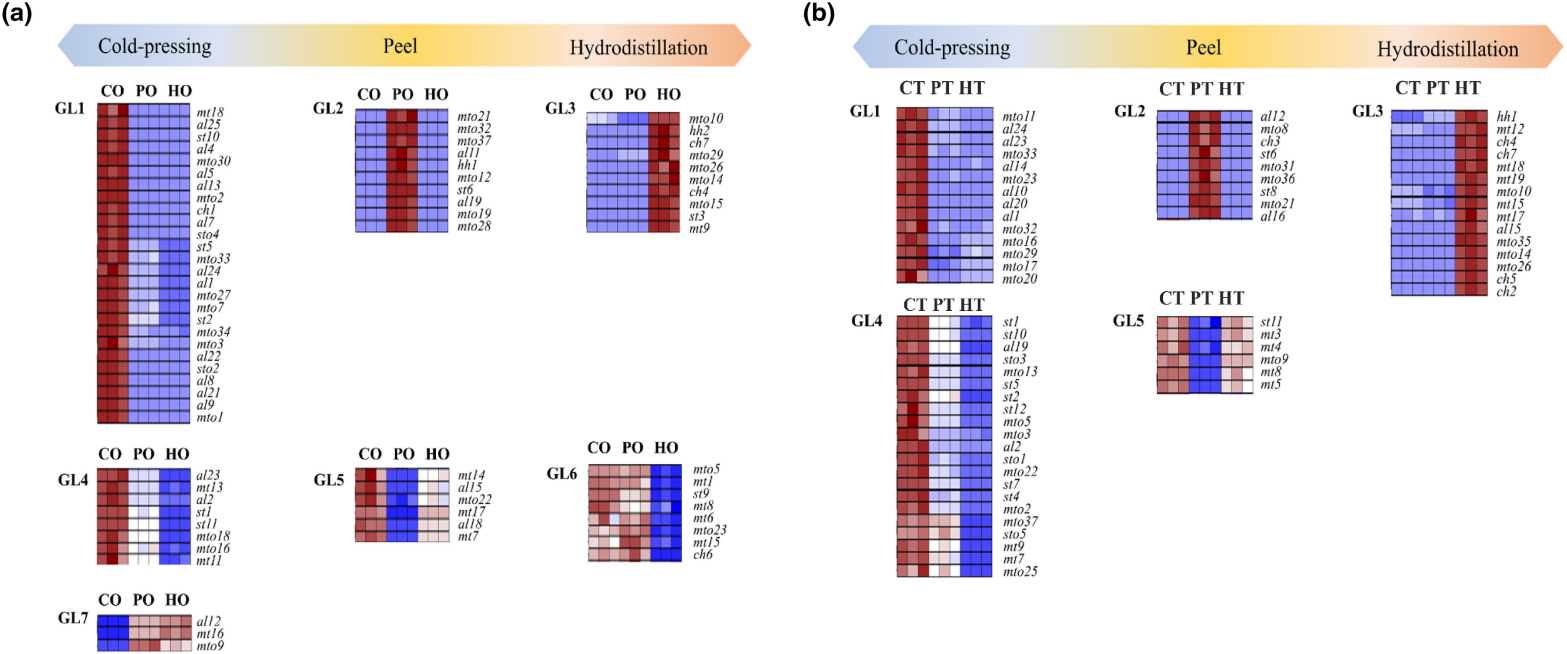
Heatmaps comparing the contents of volatile compounds depending on different extractions for essential oil. Red colors indicate that volatile compound levels were higher than mean levels, while blue colors indicate that volatile compound levels were lower than mean levels. Sample abbreviations are shown below; HM, tangor essential oil extracted by hydrodistillation; CM, tangor essential oil extracted by cold pressing; CO, orange essential oil extracted by cold pressing; HO, orange essential oil extracted by hydrodistillation; PM, the tangor peel; PO, the orange peel.

In Figure 3, a heatmap is shown to identify the variation in each volatile compound derived from orange (Figure 3a) and tangor (Figure 3b) across the essential oil extraction methods. The samples were divided into several groups based on the expression characteristics of the volatile compounds. GL1, GL4, and GL7 represent the group that showed distinctive changes in essential oil extracted using CP compared to the peel and the essential oil extracted using HD. GL1 and GL4 represent relatively up‐expressed volatile compounds compared to others (the peels and HD essential oil), while GL7 showed relatively down‐expressed volatile compounds in the CP essential oil. GL2 and GL5 indicate peel‐specific volatile compounds. GL2 contained a group of volatile compounds that were either detected only in the peel or had a relatively higher content than the other samples. GL5 contained a group of volatile compounds that were not detected in the peel or had a relatively lower content than the essential oils. GL3 and GL6 represent the volatile compounds that were either up‐ or down‐expressed or not detected in the essential oils extracted using HD. In the results, aliphatic hydrocarbons, such as fatty acids composed of six or more carbons and their oxygenated derivatives, were mainly observed in the CP extract. Nonanoic acid and hexanal were detected in high concentrations of the essential oils extracted using CP both orange and tangor. Hexanal, which is formed from linoleic acid via the lipoxygenase pathway (Gardner, 1995), is an important volatile compound that contributes to a green odor note (Gardner, 1995). It has been utilized in food and fragrance products to give fresh organoleptic note (Rouhi, 1999). In particular, oxidized fatty acids, such as 1‐hexanol, (Z)‐3‐hexen‐1‐ol, (E)‐2‐hexen‐1‐ol, hexanal, (Z)‐2‐hexen‐1‐ol, and 1‐decanol, were highly expressed compared to the orange peel and HD orange essential oil. Meanwhile, p‐cymene, γ‐terpinene, 4‐terpineol, 4‐vinylguaiacol, (E)‐carveol, cryptone, and 1,4‐dimethyl‐4‐acetylcyclohexene were highly expressed in both HD essential oils than CP essential oil. 4‐Terpineol and 4‐vinylguaiacol are potential off‐flavor markers in concentrated orange juice heated during processing (Mastello et al., 2015). 4‐Terpineol is derived from acid‐catalyzed degradation of limonene by thermal induce (Cheng et al., 2022).

In summary, the volatile compounds that were identified to represent the characteristics of orange and the tangor were explained in terms of the changes observed depending on the extraction method. Table 1 lists the volatile compounds that contribute to the distinction between the orange and the tangor. These volatile compounds were classified based on the groups shown in Figure 3. The odor description of each volatile compound was based on previous studies (Cuevas‐Glory et al., 2020; Li et al., 2016; Neiens & Steinhaus, 2018; Yan et al., 2023; Zhang et al., 2022). In terms of orange, hexanal (fatty‐green), 1‐hexanol (winey, slight fatty‐fruity), 2‐hexenal (green, fruity), (E)‐geraniol (sweet, rose‐like odor), nerol acetate (sweet fruity), (E)‐ocimene (sweet, herbal), linalool (floral‐woody, citrus), and β‐citral (citrus) showed highly up‐expressed in the CP essential oil (GL1 & 4). In the HD essential oil, p‐cymene (citrus, lemon‐like) increased (GL3), while δ‐carene (fresh, sweet), valencene (orange‐like odor), β‐myrcene (balsamic‐herbaceous), α‐terpinolene (sweet, piney), and benzenemethanol (sweet, almond fruity) decreased (GL6).

With regard to tangor, caryophyllene oxide (dry, woody), β‐elemene (woody, turpentine‐like), perilla aldehyde (spicy, fatty, herbal), α‐phellandrene (citrusy), octanoic acid (fatty, rancid), α‐sinensal (orange‐like), caryophyllene (woody, spicy), (E,E)‐α‐farnesene (citrus, green vegetative), spathulenol (honey), carveol (caraway‐like), α‐copaene (woody, spicy), 2,6‐octadiene, 2,6‐dimethyl, β‐cubebene (green, herbal), decanal (orange, sweet), calamenene (clove, herbaceous), dihydrocarvone (spearmint‐like), limonene‐1,2‐diol (grapefruit‐like), carvone (caraway odor), β‐farnesene (citrus, woody), (E)‐limonene oxide (sweet, floral), (E)‐2‐cyclohexen‐1‐ol, 1‐methyl‐4‐(1‐methyletheny), and (E)‐sabinene hydrate increased in the CP essential oil (GL 1&4). Otherwise, terpinene‐4‐ol (greasy, musty), α‐naginatene (lemon‐like), perillene (woody floral, citrus), γ‐terpinene (terpene‐like) increased in HD essential oil (GL3).

**FIGURE 4:**
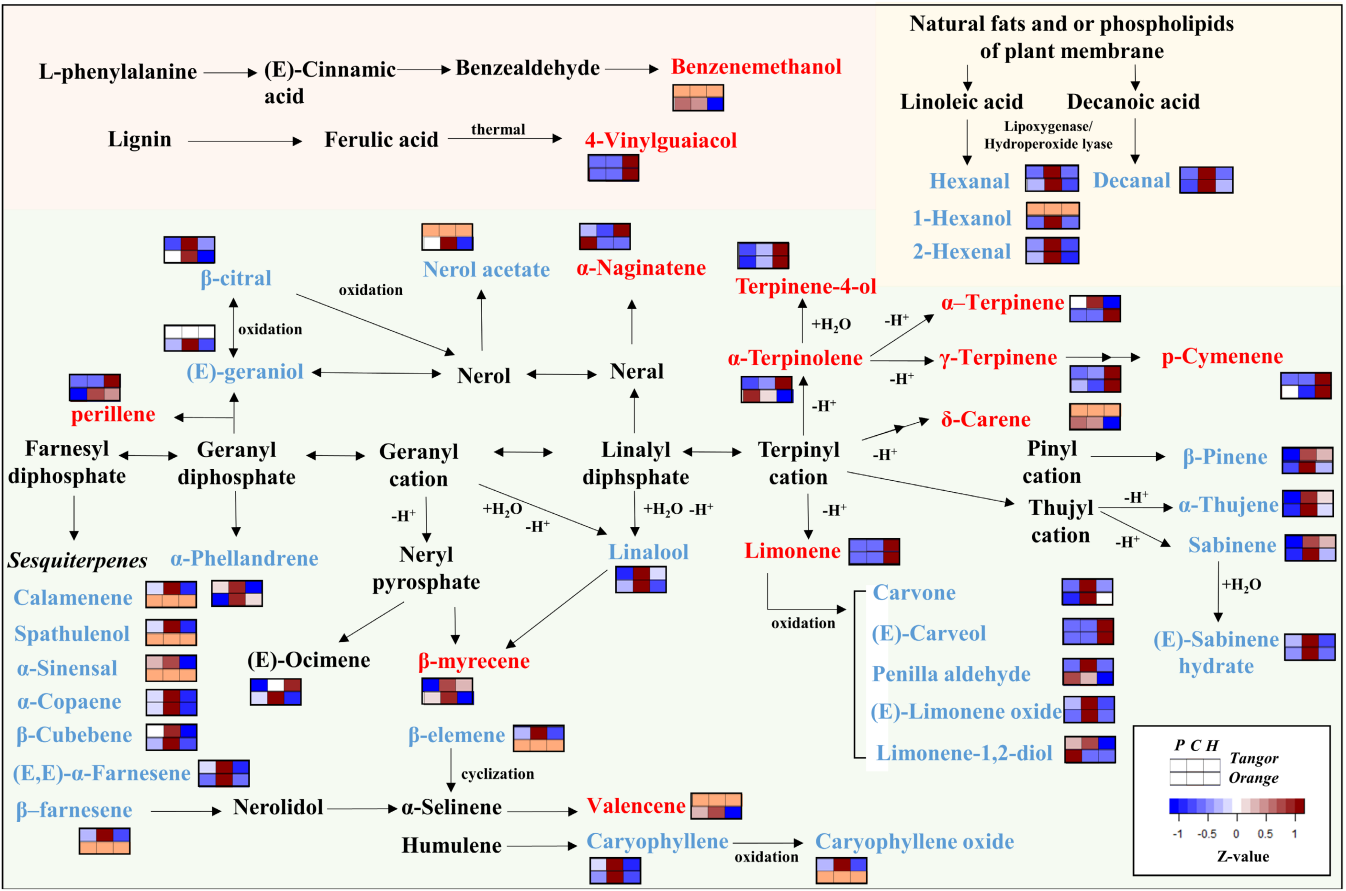
The simplified pathways related to main volatile compounds, which are contributing to the characteristics of the orange and tangor. Fold change of each volatile compound is shown using the following colors: red, increased; blue, decreased.

To summarize, the simplified pathways of the main volatile compounds in the orange and tangor are reconstructed in Figure 4 based on previous studies (Behr & Johnen, 2009; Christianson & Blank, 2020; Davis & Croteau, 2000; Moerkercke et al., 2009; Srividya et al., 2015). The changes in the volatile compounds, which were determined to contribute to the characteristics of the orange and tangor, are also expressed as colors in the figure. Based on these results, sesquiterpenes, oxygenated monoterpenes, and fatty acid derivatives (aldehydes and alcohols) were highly related to CP, while some volatile compounds derived from terpinyl cation, limonene, and 4‐vinylguaiacol were correlated with HD. In particular, the amounts of α‐terpinolene, α‐terpinene and (E)‐ocimene differed among citrus species. The reason for this was not elucidated; however, it might be explained by the different statuses of the peel (the size of oil pore, distribution on the surface, etc.) affecting the excretion of volatile compounds from the oils. In this study, limonene content increased when HD was applied. Siddiqui et al. (2022) also reported that higher temperatures improved the extraction of limonene from orange peels.

Although the results based on the extraction methods showed a similar trend, the increased or decreased amounts of some volatile compounds varied among the citrus species. For example, terpinene‐4‐ol, which is known to have an off‐flavor in orange juice (Averbeck & Schieberle, 2011), was highly increased in HD essential oil due to thermal treatment. However, the increase in the ratio induced by HD was much higher in the orange (fold change = 3.7) than in the tangor (fold change = 2.7). Another off‐flavor compound, 4‐vinylguiacol, was also 3.6 times higher in the orange essential oil than in the tangor essential oil under HD condition. Based on these results, it is necessary to consider not only the characteristics of the extraction method but also the chemical composition of citrus species.

## 4 CONCLUSION

In this study, the impacts of CP and HD, which are commonly used in the industrial field, on the formation of volatile compounds in the orange and tangor were investigated. Sesquiterpenes, oxygenated monoterpenes, and fatty acid derivatives were highly related to the essential oils prepared by CP, while terpinyl cation derivatives, limonene, and 4‐vinylguaiacol increased in the essential oils prepared using HD. On the other hand, some volatile compounds were determined by citrus species rather than by extraction methods. Therefore, to understand the effect of extraction methods on essential oils, comprehensive considerations, including the direction and degree of variation in the composition of volatile compounds, are required. In addition, this study can be expanded to explore other extraction methods or evaluate the sensory impact of the volatile compound differences in the CEOs. These various approaches will be efficiently employed to establish strategies for obtaining optimal citrus essential oils, especially that of the tangor.

## AUTHOR CONTRIBUTIONS

**Min Kyung Park:** Conceptualization (equal); data curation (equal); formal analysis (equal); methodology (equal); validation (equal); writing – original draft (equal); writing – review and editing (equal). **Ji Yoon Cha:** Formal analysis (equal); writing – original draft (equal). **Min‐Cheol Kang:** Formal analysis (equal); validation (equal). **Hae Won Jang:** Validation (equal); writing – original draft (equal). **Yun‐Sang Choi:** Conceptualization (equal); investigation (equal); supervision (equal); supervision (equal); writing – original draft (equal); writing – original draft (equal); writing – review and editing (equal); writing – review and editing (equal).

## FUNDING INFORMATION

This study was funded by Korea Food Research Institute, Grant/Award Number: E0211200‐03.

## CONFLICT OF INTEREST STATEMENT

The authors declare that they have no conflict of interest.

## ETHICS STATEMENT

This study does not involve any human or animal testing.

## Supporting information


Data S1

## Data Availability

The data supporting the findings of this study are available from the corresponding author upon reasonable request.
