# Supplementary material for: The effects of different extraction methods on essential oils from orange and tangor: From the peel to the essential oil
Source: Food Sci Nutr. 2023 Oct 25;12(2):804–14. doi: 10.1002/fsn3.3785 (PMC10867503; doi:10.1002/fsn3.3785)
Supplement: Supplementary file 1 — Data S1 [file FSN3-12-804-s001.docx]

**Supporting Information**

**Title: The effects of different extraction methods on essential oils from orange and tangor: From the peel to the essential oil**

**Running title: Flavors from citrus peel and essential oil**

Min Kyung Park^1^, Ji Yoon Cha^1^, Min-Cheol Kang^1^, Hae Won Jang^2^, Yun-Sang Choi^1,*^

**Table S1.** Volatile compounds derived from the citrus peels and the essential oils

| No^.a^ | Volatile compound | RI^b^ | Content (Mean ± standard deviation)^c^ | | | | | | ID^e^ |
| --- | --- | --- | --- | --- | --- | --- | --- | --- | --- |
|  |  |  | PT^d^ | CT | HT | PO | CO | HO |  |
| ***Monoterpene hydrocarbons*** | |  |  |  |  |  |  |  |  |
| mt1 | α-Pinene | 1,015 | 74.98±3.27a^f^ | 157.31±6.99b | 162.89±0.84b | 111.69±6.85b | 120.87±3.52c | 65.29±1.00a | A |
| mt2 | α-Thujene | 1,022 | 4.37±0.16a | 7.93±0.54c | 6.47±0.08b | 1.09±0.09a | 2.13±0.07c | 1.44±0.03b | B |
| mt3 | Camphene | 1,057 | ND^g^a | 0.97±0.16b | 0.86±0.08b | NDa | NDc | NDb | A |
| mt4 | β-Pinene | 1,101 | 29.35±5.91a | 72.97±6.87c | 61.18±1.29b | 1.87±0.15a | 8.16±0.27c | 3.72±0.73b | A |
| mt5 | Sabinene | 1,118 | 247.06±7.34a | 615.53±18.57c | 506.25±32.59b | 37.71±0.27a | 171.76±7.68c | 78.88±3.80b | A |
| mt6 | delta-Carene | 1,143 | NDa | NDa | NDa | 98.11±3.66b | 94.65±14.63b | 59.51±2.70a | A |
| mt7 | α-Phellandrene | 1,159 | 2.43±0.43b | 4.23±0.17c | NDa | NDa | 11.28±0.68c | 7.29±0.15b | A |
| mt8 | β-Myrcene | 1,164 | 185.31±2.15a | 395.56±4.48c | 345.28±41.38b | 340.16±8.53b | 420.33±23.14c | 201.8±40.09a | B |
| mt9 | α-Terpinene | 1,173 | 4.19±0.25b | 7.54±0.62c | NDa | NDa | NDa | 3.43±0.12b | A |
| mt10 | Limonene | 1,201 | 5986.86±92.43a | 9642.91±962.27b | 10876.98±57.33c | 9409.84±897.99a | 8814.53±447.45a | 5957.19±128.38b | A |
| mt11 | β-Phellandrene | 1,205 | 20.76±4.56a | 41.23±1.32b | 22.79±39.48c | 35.45±0.53b | 48.6±4.69c | 23.96±1.30a | A |
| mt12 | gamma-Terpinene | 1,240 | 6.89±0.10a | 10.05±0.74b | 34.54±0.88c | 2.18±0.07b | 1.39±0.11a | 4.23±0.09c | B |
| mt13 | (E)-Ocimene | 1,250 | 0.79±0.02a | 1.97±0.16b | 3.01±0.15c | 4.63±0.18b | 8.14±0.30c | 2.48±0.16a | A |
| mt14 | m-Cymene | 1,266 | 1.40±0.22a | 3.41±0.26b | 4.10±0.29c | 0.39±0.03a | 1.03±0.16c | 0.74±0.02b | A |
| mt15 | α-Terpinolene | 1,277 | 3.46±0.38a | 4.55±0.27b | 11.89±0.46c | 16.28±0.92c | 13.51±0.59b | 8.95±0.85a | B |
| mt16 | Neoalloocimene | 1,394 | NDa | NDa | NDa | 0.07±0c | NDa | 0.06±0.00b | B |
| mt17 | Perillene | 1,440 | 0.90±0.07b | 0.90±0.02a | 1.71±0.13c | NDa | 0.11±0.01c | 0.09±0.00b | B |
| mt18 | p-Mentha-1,5,8-triene | 1,445 | NDa | NDa | 0.63±0.02b | NDa | 1.31±0.22b | NDa | C |
| mt19 | p-Cymenene | 1,457 | NDa | NDa | 14.62±0.39b | 1.14±0.07b | NDa | 1.67±0.22c | B |
| ***Oxygenated monoterpenes*** | |  |  |  |  |  |  |  |  |
| mto1 | (Z)-Limonene oxide | 1,458 | NDa | NDa | NDa | NDa | 2.11±0.13b | NDa | B |
| mto2 | (E)-Limonene oxide | 1,474 | 7.50±0.28b | 21.28±1.99c | 1.67±0.18a | NDa | 11.01±0.25b | NDa | B |
| mto3 | (E)-Sabinene hydrate | 1,478 | 1.33±0.03b | 2.15±0.20c | 1.12±0.01a | 0.26±0.01a | 1.76±0.25b | NDa | C |
| mto4 | Citronellal | 1,490 | 0.82±0.07a | 3.98±0.17b | 4.61±0.23c | NDa | NDa | NDa | B |

**Table S1.** Cont.

| No^.a^ | Volatile compound | RI^b^ | Content (Mean ± standard deviation)^c^ | | | | | | ID^e^ |
| --- | --- | --- | --- | --- | --- | --- | --- | --- | --- |
|  |  |  | PT^d^ | CT | HT | PO | CO | HO |  |
| ***Oxygenated monoterpenes*** | |  |  |  |  |  |  |  |  |
| mto5 | Citronella | 1,494 | 4.15±0.28b | 14.03±2.10c | NDa | 5.36±0.11c | 3.93±0.05b | 1.55±0.14a | B |
| mto6 | Pinocamphone | 1,555 | NDa | 2.80±0.15c | 1.10±0.06b | NDa | NDa | NDa | C |
| mto7 | Linalool | 1,563 | 9.13±0.25a | 57.27±0.25c | 29.72±1.59b | 24.38±1.15b | 30.21±0.89c | 12.71±0.25a | A |
| mto8 | Pinocarvone | 1,573 | 0.03±0.00b | NDa | NDa | NDa | NDa | NDa | B |
| mto9 | Isopulegol | 1,580 | NDa | 0.91±0.07c | 0.77±0.01b | 0.39±0.01c | NDa | 0.19±0.02b | B |
| mto10 | 4-Terpineol | 1,609 | 1.55±0.21a | 4.71±0.28b | 17.31±1.47c | 0.51±0.01a | 1.63±0.25b | 4.34±0.01c | A |
| mto11 | Dihydrocarvone | 1,614 | 1.27±0.08a | 7.12±0.30b | 0.92±0.12a | 0.20±0.01a | 0.46±0.02b | 0.21±0.01a | A |
| mto12 | p-Menth-1-en-9-al | 1,621 | 0.22±0.02a | 3.76±0.05c | 1.33±0.08b | 0.25±0.01b | NDa | NDa | C |
| mto13 | (E)-2-Cyclohexen-1-ol, 1-methyl-4-(1-methyletheny) | 1,635 | 2.89±0.1b | 8.06±0.58c | NDa | NDa | NDa | NDa | C |
| mto14 | Cryptone | 1,671 | NDa | NDa | 1.59±0.08b | NDa | NDa | 0.16±0.03b | B |
| mto15 | (Z)-p-Menth-2,8-dienol | 1,676 | NDa | NDa | NDa | NDa | NDa | 0.52±0.02b | C |
| mto16 | β-Citral | 1,687 | NDa | 3.28±0.18c | 0.45±0.04b | 2.25±0.26b | 2.50±0.29b | 0.71±0.08a | A |
| mto17 | Terpineol | 1,706 | 3.93±0.29a | 14.41±0.31c | 6.34±0.03b | 4.68±0.43a | 6.98±0.59b | 4.05±0.30a | A |
| mto18 | Nerol acetate | 1,730 | NDa | NDa | NDa | 0.63±0.02b | 0.95±0.06c | NDa | A |
| mto19 | Citral | 1,735 | NDa | NDa | NDa | 3.94±0.15b | NDa | NDa | A |
| mto20 | Carvone | 1,743 | 34.07±1.33a | 100.4±19.53b | 40.41±1.87a | NDa | 6.04±0.14c | 2.71±0.02b | A |
| mto21 | Geranyl acetate | 1,760 | 4.26±0.25b | NDa | NDa | 0.36±0.03b | NDa | NDa | A |
| mto22 | β-Citronellol | 1,773 | 6.76±0.38b | 18.58±0.47c | 2.89±0.43a | 5.17±0.35a | 5.97±0.32b | 4.85±0.22a | B |
| mto23 | Perilla aldehyde | 1,787 | 3.11±0.43a | 12.22±0.39b | 3.22±0.13a | 0.75±0.03c | 0.46±0.02b | 0.17±0.02a | B |
| mto24 | Gamma-Geraniol | 1,794 | NDa | NDa | NDa | 0.06±0.00b | 0.18±0.03c | 0.06±0.00a | A |
| mto25 | (Z)-Geraniol | 1,808 | 0.64±0.06b | 0.91±0.14c | 0.20±0.01a | 3.57±0.18a | 9.01±0.51b | 3.58±0.27a | A |
| mto26 | (E)-Carveol | 1,841 | NDa | NDa | 12.03±0.60b | NDa | NDa | 1.73±0.33b | B |

**Table S1.** Cont.

| No^.a^ | Volatile compound | RI^b^ | Content (Mean ± standard deviation)^c^ | | | | | | ID^e^ |
| --- | --- | --- | --- | --- | --- | --- | --- | --- | --- |
|  |  |  | PT^d^ | CT | HT | PO | CO | HO |  |
| ***Oxygenated monoterpenes*** | |  |  |  |  |  |  |  |  |
| mto27 | trans-Geraniol | 1,854 | NDa | NDa | NDa | 2.18±0.03b | 6.58±0.36c | NDa | B |
| mto28 | Limonen-10-yl acetate | 1,860 | NDa | NDa | NDa | 0.31±0.01b | NDa | NDa | C |
| mto29 | Carveol | 1,872 | 2.31±0.27a | 8.92±0.19c | 3.60±0.32b | 0.16±0.01b | NDa | 0.86±0.14c | B |
| mto30 | (Z)-Carveol | 1,875 | NDa | NDa | NDa | NDa | 1±0.01b | NDa | B |
| mto31 | Perillyl Acetate | >1,900 | 0.06±0.01b | NDa | NDa | NDa | NDa | NDa | C |
| mto32 | Piperitenone | >1,900 | 0.23±0.07a | 1.41±0.18b | 0.28±0.01a | 0.02±0b | NDa | NDa | C |
| mto33 | p-Menth-1-en-9-ol | >1,900 | 0.16±0.05a | 0.74±0.06b | 0.14±0.00a | 0.15±0.01b | 0.24±0.01c | 0.06±0.00a | C |
| mto34 | Perilla alcohol | >1,900 | 0.20±0.01a | 0.71±0.03c | 0.35±0.02b | 0.14±0.01b | 0.29±0.02c | 0.08±0.01a | C |
| mto35 | p-Mentha-1,4-dien-7-ol | >1,900 | NDa | NDa | 0.05±0.00b | NDa | NDa | NDa | C |
| mto36 | Cuminol | >1,900 | 0.05±0.01b | NDa | NDa | NDa | NDa | NDa | C |
| mto37 | Limonene-1,2-Diol | >1,900 | 1.49±0.07b | 1.89±0.10c | NDa | 0.24±0.01b | NDa | NDa | C |
| ***Sesquiterpene hydrocarbons*** | | |  |  |  |  |  |  |  |
| st1 | α-Copaene | 1,502 | 2.70±0.15b | 5.71±0.03c | 0.37±0.02a | 1.26±0.05b | 2.30±0.07c | 0.43±0.02a | B |
| st2 | β-Cubebene | 1,545 | 1.50±0.12b | 2.79±0.28c | NDa | 0.28±0.01b | 0.81±0.05c | 0.06±0.00a | B |
| st3 | β-Gurjunene | 1,594 | NDa | NDa | NDa | NDa | NDa | 0.40±0.01b | B |
| st4 | β-elemene | 1,598 | 0.17±0.03b | 0.71±0.05c | NDa | NDa | NDa | NDa | A |
| st5 | Caryophyllene | 1,600 | 1.48±0.07b | 3.95±0.09c | NDa | 0.95±0.01b | 2.59±0.13c | 0.56±0.02a | B |
| st6 | Alloaromadendrene | 1,645 | 0.32±0.07b | NDa | NDa | 0.19±0.00b | NDa | NDa | B |
| st7 | β-Farnesene | 1,677 | 8.61±0.33b | 30.50±0.32c | NDa | NDa | NDa | NDa | A |
| st8 | Eremophilene | 1,722 | 1.93±0.15b | NDa | NDa | NDa | NDa | NDa | B |
| st9 | Valencene | 1,729 | NDa | NDa | NDa | 3.08±0.13b | 4.09±0.15c | 0.55±0.02a | B |
| st10 | (E,E)-α-Farnesene | 1,752 | 2.38±0.3b | 5.45±0.01c | NDa | NDa | 0.25±0.03b | NDa | B |
| st11 | delta-Cadinene | 1,756 | 1.65±0.21a | 2.88±0.21b | 2.70±0.19b | 0.42±0.01b | 0.94±0.04c | NDa | B |

**Table S1.** Cont.

| No^.a^ | Volatile compound | RI^b^ | Content (Mean ± standard deviation)^c^ | | | | | | ID^e^ |
| --- | --- | --- | --- | --- | --- | --- | --- | --- | --- |
|  |  |  | PT^d^ | CT | HT | PO | CO | HO |  |
| ***Sesquiterpene hydrocarbons*** | | |  |  |  |  |  |  |  |
| st12 | Calamenene | 1,832 | 0.05±0.00b | 0.14±0.02c | NDa | NDa | NDa | NDa | B |
| ***Oxygenated sesquiterpenes*** | |  |  |  |  |  |  |  |  |
| sto1 | Caryophyllene oxide | >1900 | 0.11±0.02b | 0.53±0.02c | NDa | NDa | NDa | NDa | C |
| sto2 | Elemol | >1900 | NDa | NDa | NDa | NDa | 0.06±0c | NDb | C |
| sto3 | Spathulenol | >1900 | 0.23±0.03b | 0.65±0.03c | NDa | NDa | NDa | NDa | C |
| sto4 | β-Sinensal | >1900 | NDa | NDa | NDa | NDa | 0.26±0.01b | NDa | C |
| sto5 | α-sinensal | >1900 | 0.09±0.01b | 0.11±0.00c | NDa | NDa | NDa | NDa | C |
| ***Cyclic or aromatic hydrocarbons*** | | |  |  |  |  |  |  |  |
| ch1 | Styrene | 1,258 | NDa | NDa | NDa | NDa | 0.51±0.02b | NDa | A |
| ch2 | 2-Methylcyclohexanol | 1,388 | NDa | NDa | 0.16±0.01b | NDa | NDa | NDa | B |
| ch3 | Benzaldehyde | 1,526 | 0.21±0.03b | NDa | NDa | NDa | NDa | NDa | B |
| ch4 | 1,4-Dimethyl-4-acetylcyclohexene | 1,528 | NDa | NDa | 0.21±0.02b | NDa | NDa | 0.05±0.00b | C |
| ch5 | 4-isopropyl-cyclohexanol | 1,647 | NDa | NDa | 0.09±0.01b | NDa | NDa | NDa | C |
| ch6 | Benzenemethanol | 1,887 | NDa | NDa | NDa | 0.14±0.03b | 0.13±0.00b | NDa | A |
| ch7 | 2-Methoxy-4-vinylphenol | >1900 | NDa | NDa | 0.29±0.02b | NDa | NDa | 0.08±0.01b | A |
| ***Aliphatic hydrocarbons*** | |  |  |  |  |  |  |  |  |
| al1 | Hexanal | 1,085 | NDa | 4.50±0.21b | NDa | 0.47±0.01b | 2.23±0.07c | NDa | A |
| al2 | 2-Hexenal | 1,217 | 0.55±0.01b | 3.56±0.2c | NDa | 2.75±0.18b | 8.67±0.55c | NDa | A |
| al3 | Octanal | 1,287 | 7.62±0.2a | 40.82±1.21c | 24.92±0.63b | 0.93±0.06a | 7.67±0.18c | 2.66±0.27b | A |
| al4 | 1-Hexanol | 1,368 | NDa | NDa | NDa | 0.27±0.02a | 5.23±0.28b | 0.08±0.02a | A |
| al5 | (Z)-3-Hexen-1-ol | 1,396 | NDa | 1.52±0.22c | 0.65±0.01b | NDa | 0.61±0.02b | NDa | B |
| al6 | Nonanal | 1,417 | 2.61±0.17a | 12.14±0.12c | 7.84±0.09b | 1.16±0.11a | 6.83±0.13c | 3.36±0.27b | A |
| al7 | (E)-2-Hexen-1-ol | 1,418 | NDa | NDa | NDa | NDa | 5.18±0.16b | NDa | B |
| al8 | (Z)-2-Hexen-1-ol | 1,427 | NDa | NDa | NDa | NDa | 0.30±0.02b | NDa | B |

**Table S1.** Cont.

| No^.a^ | Volatile compound | RI^b^ | Content (Mean ± standard deviation)^c^ | | | | | | ID^e^ |
| --- | --- | --- | --- | --- | --- | --- | --- | --- | --- |
|  |  |  | PT^d^ | CT | HT | PO | CO | HO |  |
| ***Aliphatic hydrocarbons*** | |  |  |  |  |  |  |  |  |
| al9 | Butanoic acid, hexyl ester | 1,431 | NDa | NDa | NDa | NDa | 0.19±0.01b | NDa | B |
| al10 | (E)-7-Tetradecene | 1,447 | NDa | 1.24±0.10b | NDa | NDa | NDa | NDa | B |
| al11 | Cosmene | 1,457 | NDa | NDa | NDa | 0.14±0.01b | NDa | NDa | B |
| al12 | Acetic acid, octyl ester | 1,485 | 0.50±0.04b | NDa | NDa | 0.54±0.01b | NDa | 0.61±0.01c | B |
| al13 | Acetic acid, 2-ethylhexyl ester | 1,487 | NDa | NDa | NDa | NDa | 1.63±0.03a | NDb | B |
| al14 | Decanal | 1,515 | 13.71±0.41a | 32.37±1.04b | 13.75±1.08a | 9.24±0.10a | 43.8±2.13c | 16.54±2.41b | A |
| al15 | 1-Octanol | 1,569 | NDa | NDa | 8.98±1.15b | 1.90±0.07a | 24.55±3.66c | 13.79±1.71b | A |
| al16 | Acetic acid, nonyl ester | 1,585 | 0.04±0.00b | NDa | NDa | NDa | NDa | NDa | B |
| al17 | (E)-2-Decenal | 1,646 | NDa | NDa | NDa | NDa | 0.32±0.03c | 0.10±0.00b | A |
| al18 | 1-Nonanol | 1,666 | NDa | NDa | NDa | NDa | 4.17±0.03c | 2.67±0.10b | A |
| al19 | 2,6-Octadiene, 2,6-dimethyl- | 1,668 | 3.01±0.23b | 6.04±0.25c | NDa | 0.69±0.02b | NDa | NDa | C |
| al20 | Dodecanal | 1,721 | NDa | 4.25±0.10b | NDa | NDa | 1.5±0.12c | 0.61±0.01b | B |
| al21 | 1-Decanol | 1,770 | NDa | NDa | NDa | NDa | 4.54±0.27b | NDa | A |
| al22 | Hexanoic acid | 1,865 | NDa | NDa | NDa | NDa | 0.57±0.02b | NDa | A |
| al23 | Octanoic acid | >1900 | 0.09±0.01b | 1.15±0.03c | NDa | 0.04±0.00b | 0.11±0.01c | NDa | A |
| al24 | Nonanoic acid | >1900 | 0.05±0.00b | 0.42±0.03c | NDa | 0.05±0.00b | 0.29±0.03c | NDa | A |
| al25 | Decanoic acid | >1900 | NDa | NDa | NDa | NDa | 0.12±0.01b | NDa | A |
| ***Heteroarmatic hydrocarbons*** | | |  |  |  |  |  |  |  |
| hh1 | α-Naginatene | 1,408 | 0.27±0.01b | NDa | 1.33±0.10c | 0.14±0.01b | NDa | NDa | A |
| hh2 | 2-Methyl-2,3-dihydrobenzofuran | 1,441 | NDa | NDa | NDa | NDa | NDa | 0.18±0.02b | C |

^a^ Numbered in the order of retention indices (RI).

^b^ RI; Retention indices were determined using n-alkanes (C_7_– C_22_)

^c^ Mean values of relative peak area to the area of internal standard ± standard deviation.

^d^ PT, the peel of tangerine; CT, the tangerine essential oil extracted by cold-pressing; HT, the tangerine essential oil extracted by hydrodistillation; PO, the peel of orange; CO, the orange essential oil extracted by cold-pressing; HO, the orange essential oil extracted by hydrodistillation.

^e^ Identification of the compounds was performed as follows: C, mass spectrum was consistent with that of W9N08 and manual interpretation (tentative identification); B, retention index and mass spectrum were consistent with those from the NIST Chemistry Webbook (tentative identification); A, retention index and mass spectrum matched with those of authentic compounds (positive identification)

^g^ Not detected.

^f^ There are significant differences (p<0.05) among each *B. subtilis* sample collected according to cultivation time determined using Duncan’s multiple range test between different times having different lowercase letters.
